# Effect of Flue Gas Desulfurization Gypsum on the Properties of Calcium Sulfoaluminate Cement Blended with Ground Granulated Blast Furnace Slag

**DOI:** 10.3390/ma14020382

**Published:** 2021-01-14

**Authors:** Danying Gao, Zhenqing Zhang, Yang Meng, Jiyu Tang, Lin Yang

**Affiliations:** 1 School of Water Conservancy Engineering, Zhengzhou University, Zhengzhou 450001, China; gdy@zzu.edu.cn (D.G.); zzq1017@gs.zzu.edu.cn (Z.Z.); 2 School of Civil Engineering, Zhengzhou University, Zhengzhou 450001, China; cy137953186@163.com (Y.M.); tjy74@zzu.edu.cn (J.T.)

**Keywords:** calcium sulfoaluminate cement, flue gas desulfurization gypsum, hydration, mechanical strength, microstructure

## Abstract

This work aims to investigate the effect of additional flue gas desulfurization gypsum (FGDG) on the properties of calcium sulfoaluminate cement (CSAC) blended with ground granulated blast furnace slag (GGBFS). The hydration rate, setting time, mechanical strength, pore structure and hydration products of the CSAC-GGBFS mixture containing FGDG were investigated systematically. The results show that the addition of FGDG promotes the hydration of the CSAC-GGBFS mixture and improves its mechanical strength; however, the FGDG content should not exceed 6%.

## 1. Introduction

Calcium sulfoaluminate cement (CSAC), which has been manufactured and used on a large scale in China since the 1970s, is considered environment-friendly with low CO_2_ emissions [1,2,3,4]. Compared with Portland cement, CSAC exhibits a shorter setting time, faster hardening, higher early strength, better durability, etc., and is widely used in various special areas of engineering, such as rapid repair engineering, ocean engineering, winter construction and so on [5,6].

The main mineral phases of CSAC clinker are ye’elimite ($C_{4}A_{3}\bar{S}$), belite ($C_{2}S$) and ferrite ($C_{4}AF$) [7]. Among them, the content of $C_{4}A_{3}\bar{S}$ usually accounts for more than 60% of the total mineral phases, and it is the greatest contributor to the performance of CSAC. For the hydration of $C_{4}A_{3}\bar{S}$, the content of gypsum directly affects the formation of hydration products, as follows [8,9,10]:

In the absence of gypsum,

(1) $$C_{4}A_{3}\bar{S}+18H\to C_{3}A\cdot C\bar{S}\cdot H_{12}+2AH_{3}$$

In the presence of gypsum, but in insufficient amounts,

(2) $$C_{4}A_{3}\bar{S}+2C\bar{S}H_{2}+34H\to C_{3}A\cdot 3C\bar{S}\cdot H_{32}+2AH_{3}$$

(3) $$C_{3}A\cdot 3C\bar{S}\cdot H_{32}\to C_{3}A\cdot C\bar{S}\cdot H_{12}+2C\bar{S}H_{2}+16H$$

If the gypsum is sufficient and calcium hydroxide (*CH*) is present,

(4) $$AH_{3}+3C\bar{S}H_{2}+3CH+20H\to C_{3}A\cdot 3C\bar{S}\cdot H_{32}$$

Then, $C_{4}A_{3}\bar{S}$ is hydrated into $C_{3}A\cdot 3C\bar{S}\cdot H_{32}$ completely:

(5) $$C_{4}A_{3}\bar{S}+8C\bar{S}H_{2}+6CH+74H\to 3\left[C_{3}A\cdot 3C\bar{S}\cdot H_{32}\right]$$

The main hydration products of $C_{4}A_{3}\bar{S}$ are ettringite ($C_{3}A\cdot 3C\bar{S}\cdot H_{32}$, AFt), monosulfoaluminate ($C_{3}A\cdot C\bar{S}\cdot H_{12}$, AFm) and aluminum hydroxide ($AH_{3}$). Usually, the Al^3+^ of $C_{4}A_{3}\bar{S}$ can be partly replaced by Fe^3+^ during the formation process of mineral phases. At that point, the hydration products also contain Fe_2_O_3_, such as $C_{3}A(F)\cdot 3C\bar{S}\cdot H_{32}$ (AFt), $C_{3}A(F)\cdot C\bar{S}\cdot H_{12}$ (AFm) and so on [1,6]. The hydration of $C_{4}AF$ is similar to that of $C_{4}A_{3}\bar{S}$. The formation of AFt determines the hydration evolution and microstructure of cement paste, and it is an essential contributor to the mechanical strength of CSAC, especially the early strength [7]. From Equations (1)–(5), it can be seen that the molar ratio of $C\bar{S}\cdot H_{2}$ to $C_{4}A_{3}\bar{S}$ (abbreviated to *M*_g/y_) is a critical parameter for the hydration of CSAC, which further determines the performance of CSAC. For *M*_g/y_ < 1.5, the CSAC shows the characteristics of rapid setting and hardening, while it is used for self-stressing when 1.5 < *M*_g/y_ < 2.5; however, it is expansive for *M*_g/y_ > 2.5 [1,7,11]. Therefore, the gypsum content plays an important role in the hydration of CSAC, and the composition of hydration products depends on the gypsum content [12]. In theory, according to Equation (5), 225.7 g of $C\bar{S}\cdot H_{2}$ is necessary for the complete hydration of 100 g of $C_{4}A_{3}\bar{S}$. However, the gypsum content should be controlled, as the amount of AFt directly affects the volume stability of CSAC [13].

In recent years, a number of supplementary cementitious materials (SCMs) have been blended with CSAC to reduce the total cost and modify the properties, such as fly ash [14,15], limestone [16], silica fume [17] and so on. In our previous work, the properties of CSAC blended with different content of GGBFS were investigated systematically [18]. The results show that the gypsum obtained from CSAC is insufficient for the hydration of the CSAC-GGBFS mixture, and additional gypsum is necessary. Furthermore, as a kind of sulfate, gypsum has the potential to excite the pozzolanic activity of GGBFS and promote its hydration. Therefore, in this work, the authors aim to investigate the effect of additional gypsum on the hydration of a CSAC-GGBFS mixture.

It is well known that the resource of natural gypsum is limited across the globe. Fortunately, flue gas desulfurization gypsum (FGDG), a by-product obtained from the desulfurization of coal-fired flue gas, supplies a potential resource of gypsum [19]. The chemical composition of FGDG is similar to that of natural gypsum, and the content of calcium sulfate dihydrate ($C\bar{S}\cdot H_{2}$) is usually more than 90%, which also contains SiO_2_, Al_2_O_3_, Fe_2_O_3_, MgO, Na_2_O, K_2_O and other impurities [19,20,21]. The annual output of FGDG is about 80 million tons in China; however, the utilization ratio is very low, and more than 50% of FGDG is disposed of as waste in industrial landfills, which not only occupies a lot of land but also pollutes the surrounding environment, including groundwater, soil, air and so on, due to the existence of contaminants (Hg, As, Se, Cl, F, etc.) [19,22]. Utilization of FGDG in the cement industry as a substitute of natural gypsum provides an effective approach to the application of FGDG.

In this work, FGDG was added in the CSAC-GGBFS blended mixture as additional gypsum, and the effect of FGDG content on the properties of the CSAC-GGBFS system was studied. This study contributes to the understanding of the hydration of this blended mixture and further improvement of its properties.

## 2. Materials and Methods

### 2.1. Raw Materials and Mix Proportion

**Figure 1:**
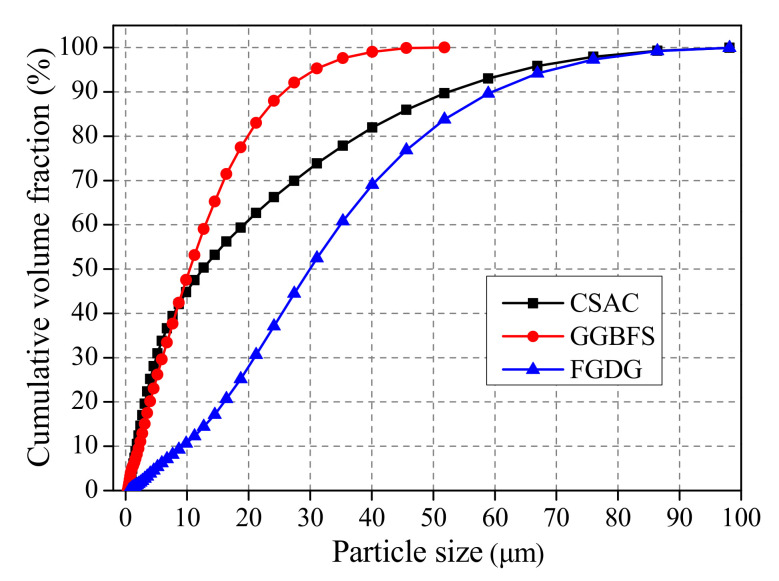
Particle size distribution of raw materials [18].

Chinese standard 42.5R CSAC (produced by Dengfeng Electric Power Group Cement Co., Ltd., Dengfeng, Henan, China), which was used in this work, was prepared with 78% clinker, 16% anhydrite and 6% limestone. Moreover, the clinker was composed with 28% C_2_S, 13.35% C_4_AF and 58.68% $C_{4}A_{3}\bar{S}$. Grade S95 GGBFS was bought in the market, and FGDG was supplied by China United Cement Anyang Co. Ltd. (Anyang, China). The chemical compositions of the raw materials are shown in Table 1, and their particle size distribution is illustrated in Figure 1. For the preparation of mortar used for measuring mechanical strength, the standard sand (in ISO 9001) was used, and the ratio of binder to sand was 1:3.

Four mix proportions of blended cement paste were designed, as shown in Table 2. According to previous research [18], the ratio of CSAC to GGBFS was invariably 8:2, and the FGDG content was 0%, 3%, 6% and 9%. The ratio of water to binder (W/B) was 0.5 in all experiments except the measurement of setting time.

### 2.2. Test Methods

The hydration heat of the mixture was measured using a TAM Air isothermal calorimeter (TA Instruments, New Castle, PA, USA), and the setting time was tested using the Vicat method, according to the Chinese standard GB/T1346-2011 [23]. The mineral phases were determined using X-ray diffraction (XRD) (X’Pert3 Powder, Malvern PANalytical, The Netherland; Cu-Kα, voltage 40 kV, current 40 mA and scanning speed 0.007 °/s). The microstructure of hardened cement paste was observed using a scanning electron microscope (SEM) (EVO HD15, ZEISS, Jena, Germany, voltage 10 kV). The pore structure was tested using mercury intrusion porosimetry (MIP) (AutoPore IV 9500), and the pores are considered to be cylindrical. The Thermogravimetric-Differential Scanning Calorimeter (TG-DSC) was conducted using a simultaneous thermal analysis instrument (SAT 449F3, NETZSCH, Selb, Germany), the heating rate was 10 °C/min, and high-purity (99.99%) nitrogen gas was used as a protective gas. For the above testing, hydration of the samples was first stopped using anhydrous ethanol, and then the samples were dried at 50 °C in an oven for three days. For the measurement of mechanical strength, mortars with dimensions of 40 mm × 40 mm × 160 mm were prepared; one group contained three specimens, which were designed according to the Chinese standard GB/T17671-1999 [24]. The test methods are the same as those illustrated in reference [18].

## 3. Results and Discussion

### 3.1. Hydration Heat

**Figure 2:**
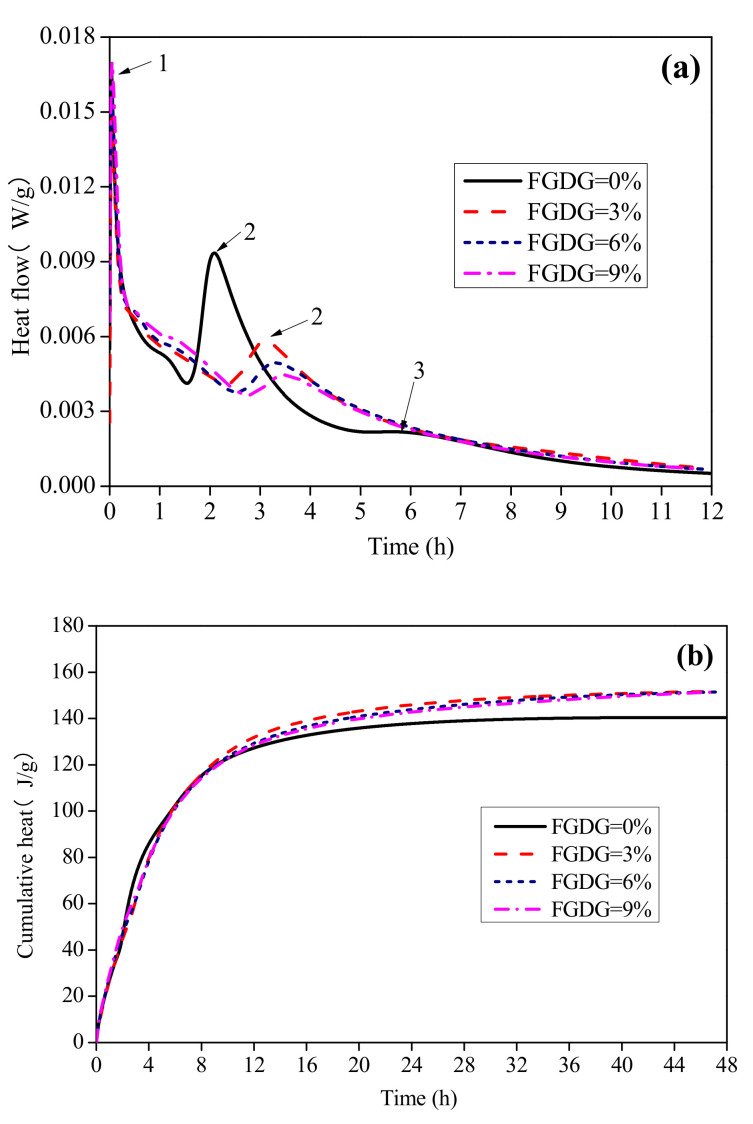
Hydration heat of CSAC-GGBFS mixtures with different FGDG content. (**a**) Heat flow, (**b**) cumulative heat.

The hydration heat flow and cumulative heat of CSAC-GGBFS cement pastes blended with different FGDG content are shown in Figure 2. For the blended cement paste without FGDG, as shown in Figure 2a, there are three peaks in the curve of heat flow: the emission of solution heat (peak 1), the main hydration reaction (peak 2) and the transformation of hydration products (peak 3) [18,25,26]. However, with the addition of FGDG, there are only two peaks in the curve of heat flow, and the third peak caused by the transformation of hydration products (Equation (3)) disappears. Additionally, the duration of the induction period is prolonged with the increase in FGDG; that is, the time to reach the main exothermal peak of the hydration reaction (Equations (1) and (2) and the hydration of C_2_S) is delayed. The figure also indicates that the height of the second peak decreases as the FGDG content increases from 0% to 9%. This illustrates that the early age hydration rate of CSAC-GGBFS can be reduced by the addition of FGDG; the greater the FGDG content, the stronger the reducing effect.

As shown in Figure 2b, the cumulative hydration heat does not significantly differ before 10 h, when the FGDG content increases from 0% to 9%. However, after 10 h, the cumulative hydration heat of cement paste blended with FGDG is increasingly higher than that without FGDG. This means that the addition of FGDG promotes the hydration of CSAC-GGBFS cement paste after 10 h. In addition, the cumulative hydration heat is similar when the cement pastes blended with 3%–9% FGDG are hydrated for 48 h.

### 3.2. Setting Time

**Figure 3:**
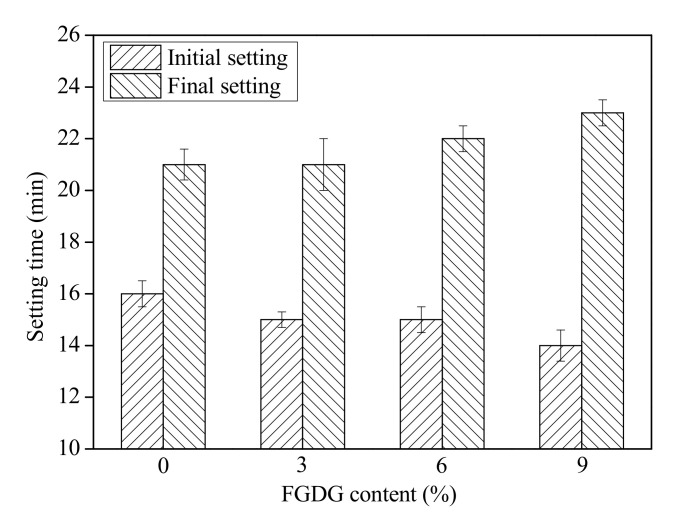
Setting time of CSAC-GGBFS mixtures blended with different FGDG content.

The setting time of CSAC-GGBFS mixtures blended with different FGDG content is shown in Figure 3. The initial setting time of blended cement paste decreases from 16 min to 14 min as the FGDG content increases from 0% to 9%; however, in contrast to the initial setting, the final setting time increases from 21 min to 23 min with increasing FGDG content. Although the addition of FGDG has some effect on the setting time of CSAC-GGBFS cement paste, the variation can be neglected, and the blended cement paste still exhibits the characteristic of rapid setting, even when additional FGDG content reaches 9%.

### 3.3. Mechanical Strength

**Figure 4:**
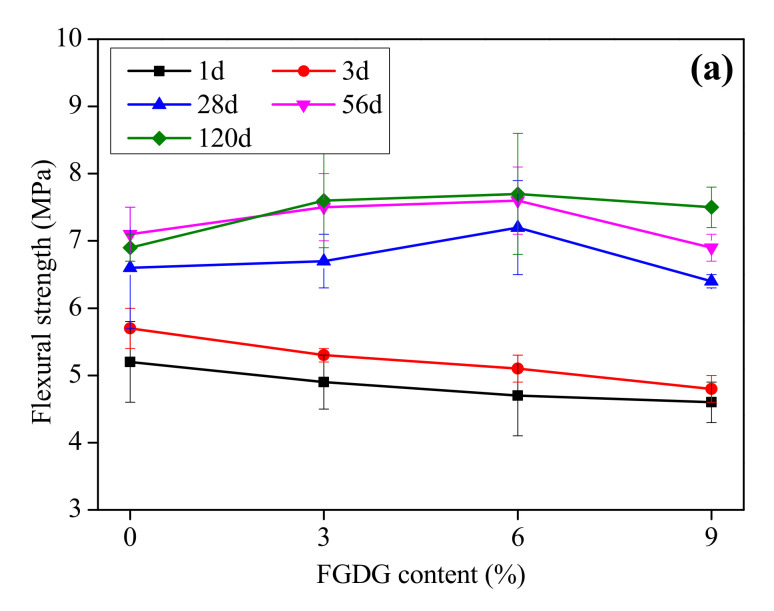

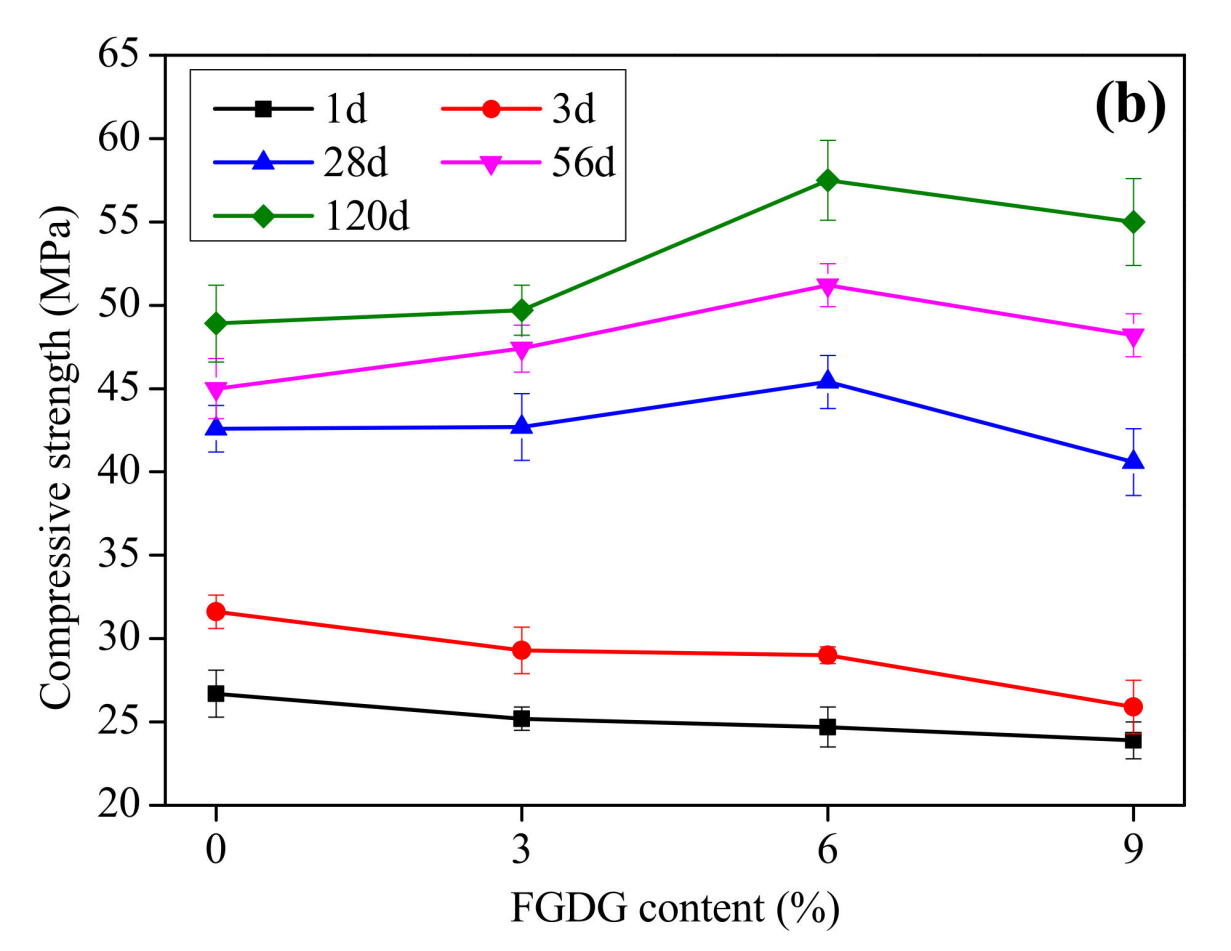
Effect of FGDG content on the mechanical strength of mortar. (**a**) Flexural strength, (**b**) compressive strength.

The mechanical strength of CSAC-GGBFS mortars blended with different FGDG content is shown in Figure 4. It can be seen that the flexural and compressive strengths of mortar develop in the same way as the FGDG increases from 0% to 9%. At 1 d and 3 d, the mechanical strength noticeably decreases with increasing FGDG content, and the flexural strength decreases by 11.5% and 15.8%, respectively; meanwhile, the compressive strength decreases by 10.5% and 18%, respectively. This is because the addition of FGDG delays the early age hydration of the CSAC-GGBFS mixture, as illustrated by the hydration heat. However, after 28 d, the flexural and compressive strength increases as the FGDG content increases from 0% to 6%, while they decrease suddenly when the FGDG content reaches 9%. According to the above results, the addition of FGDG can promote the later age hydration of CSAC-GGBFS. Moreover, with the increase in FGDG, the amount of AFt increases due to the hydration reactions of Equations (2) and (4), which is the main contributor to the mechanical strength of the CSAC-GGBFS system [7]. Furthermore, as a sulfate, FGDG can excite the pozzolanic activity of GGBFS, and the second hydration reaction occurs. However, when the FGDG content reaches 9%, the amount of AFt becomes excessive, resulting in expansion and micro-cracks, and the flexural and compressive strengths of mortar are reduced. It can be seen that the amount of FGDG has a significant effect on the mechanical strength development of the CSAC-GGBFS blended mixture.

### 3.4. Pore Structure

**Figure 5:**
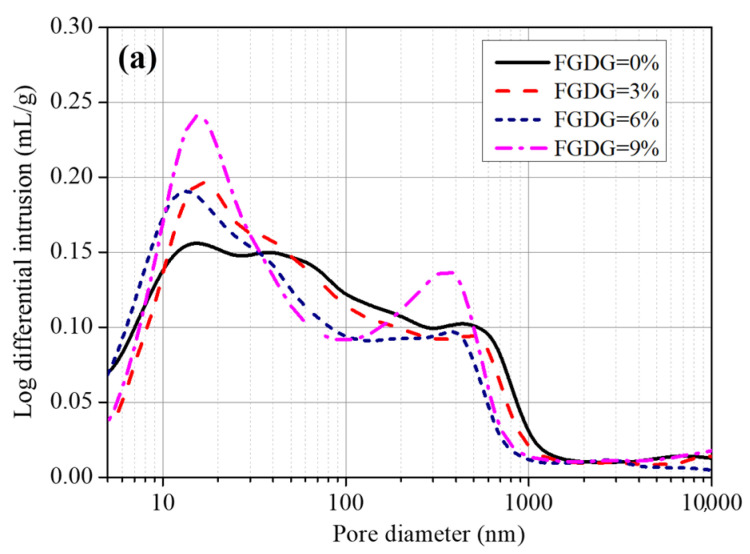

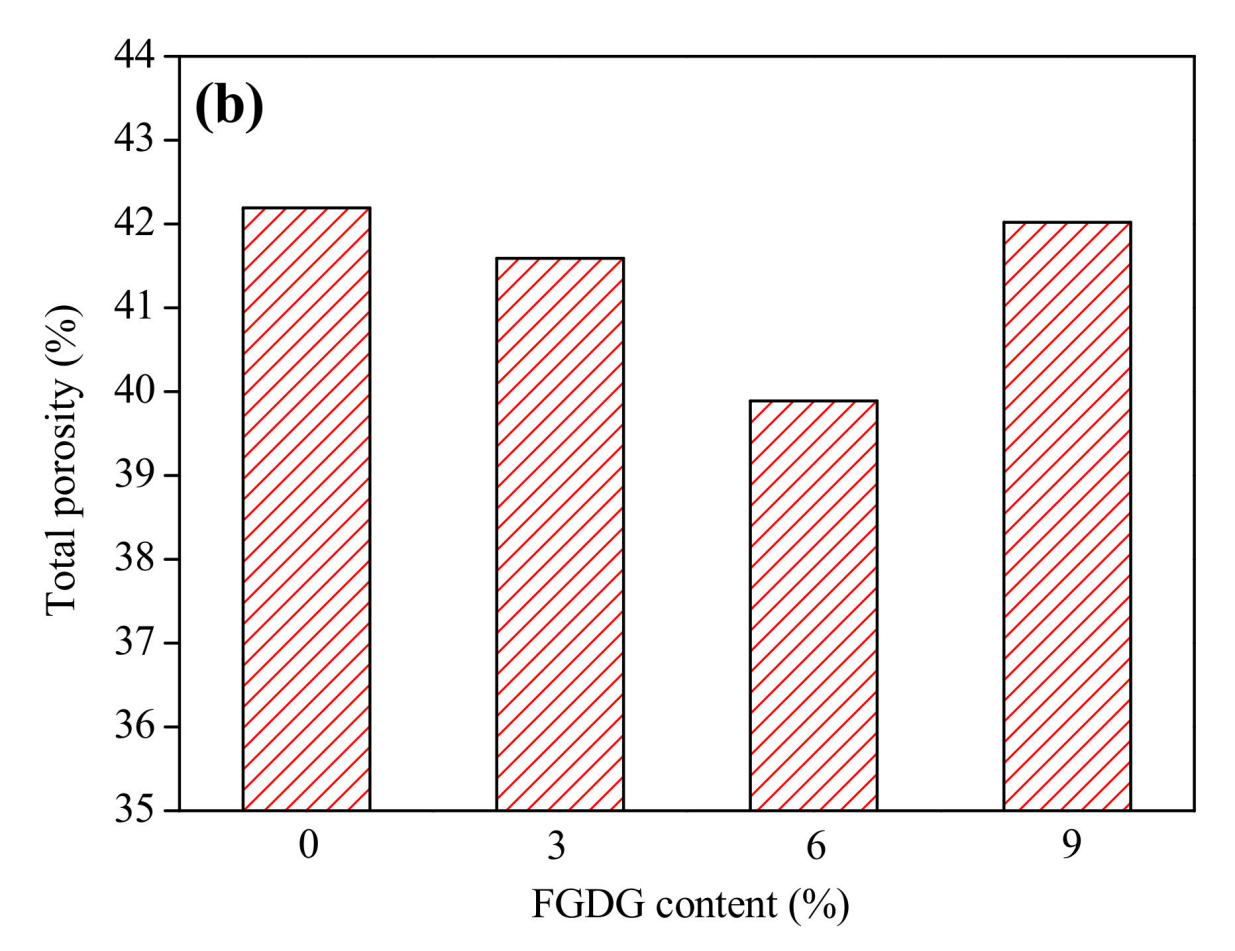
Pore structure of CSAC-GGBFS mixtures blended with different FGDG content hydrated for 120 d. (**a**) Pore size distribution, (**b**) total porosity.

The pore structure of CSAC-GGBFS mixtures blended with different FGDG content hydrated for 120 d is shown in Figure 5. As shown in Figure 5a, the pore size ranges of cement pastes blended with different FGDG content are similar to each other, 5–1000 nm in total. However, the pore size distribution shifts to the left (smaller pores) when the FGDG content increases from 0% to 6%; in other words, the number of large pores (>40 nm) decreases while the number of small pores (<40 nm) increases. Meanwhile, the total porosity decreases from 42.2% to 39.9% as the FGDG content increases from 0% to 6%, as shown in Figure 5b. However, when the FGDG content increases to 9%, not only does the number of small pores (<30 nm) increase, but the number of large pores (>100 nm) also increases, and then the total porosity increases again. The addition of FGDG promotes the hydration of CSAC-GGBFS cement paste and the generation of more hydration products (AFt and C–S–H gel), which fill the microstructure and reduce the total porosity. However, when the FGDG content increases to 9%, the excessive AFt results in cracking due to its expansion [22] as well as in an increase in the number of large pores, which, in turn, increases the total porosity.

From the above results, it can be seen that an appropriate quantity of FGDG is helpful in refining the pore size and reducing the total porosity of the CSAC-GGBFS mixture, which is the direct reason that the flexural strength and compressive strength increase as the FGDG content increases from 0% to 6%.

### 3.5. Hydration Product Analysis

#### 3.5.1. X-ray Diffraction (XRD)

**Figure 6:**
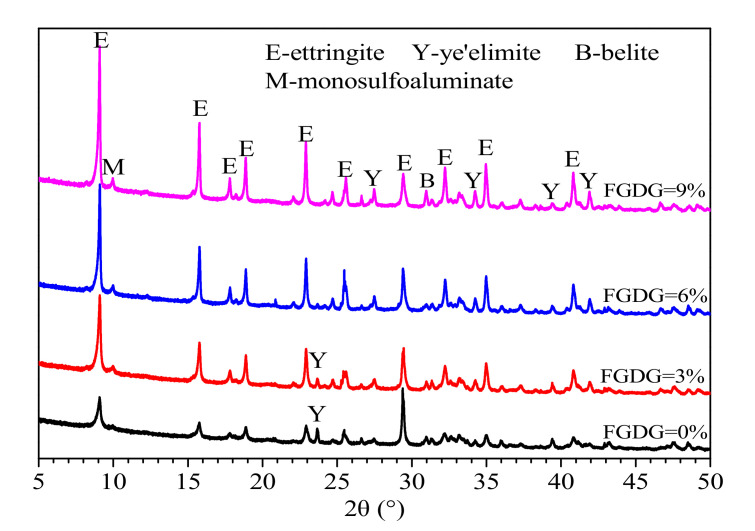
XRD patterns of CSAC-GGBFS mixtures blended with different FGDG content hydrated for 120 d.

Figure 6 shows the XRD patterns of CSAC-GGBFS mixtures blended with different FGDG content hydrated for 120 d. It is clear that the main mineral phases are AFt, AFm, unhydrated $C_{4}A_{3}\bar{S}$ and $C_{2}S$. The intensity of the AFt diffraction peak increases noticeably as the FGDG content increases from 0% to 9%. This indicates that the amount of AFt increases with increasing FGDG content, as the same equipment and test parameters were used [27]. This result illustrates that the addition of FGDG promotes the hydration of CSAC-GGBFS cement paste, and more Aft is generated due to the hydration reactions of Equations (2) and (4).

**Figure 7:**
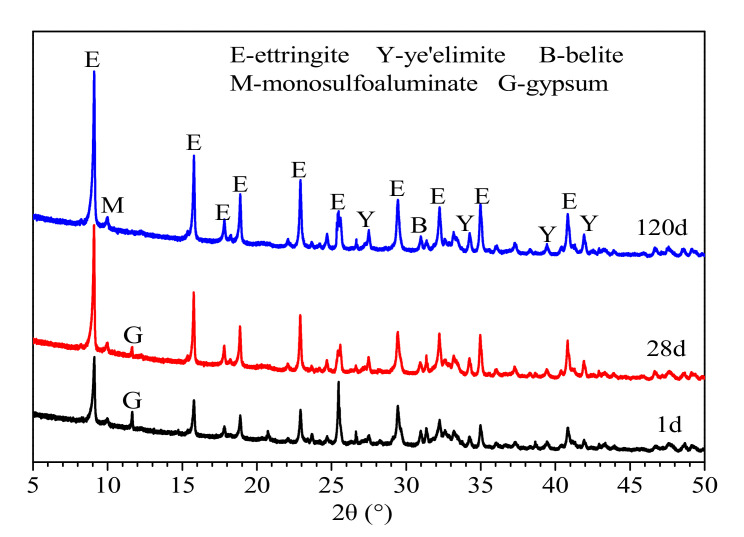
XRD patterns of CSAC-GGBFS mixture blended with 6% FGDG hydrated for different time intervals.

Figure 7 shows the XRD patterns of the CSAC-GGBFS mixture blended with 6% FGDG hydrated for different periods of time. It can be seen that the diffraction peak of gypsum can be detected when the cement paste is hydrated for 1 d and 28 d; however, it disappears at 120 d. That is to say, the gypsum is not consumed completely until the cement paste is hydrated for 120 d. Furthermore, the amount of AFt increases significantly as the curing time increases from 1 d to 120 d. It can also be seen that the mineral phase of AFm still exists at 120 d, which indicates that the mineral phases of $C_{4}A_{3}\bar{S}$ are not hydrated into AFt completely.

According to Figure 6 and Figure 7, as the hydration product of C_2_S, CH does not exist in the cement paste since it is consumed by the secondary hydration reaction (Equation (4)). In addition, the phases of AH3 and C–S–H gel are not detected by XRD, as they are amorphous; however, their occurrence can be determined by thermal analysis.

#### 3.5.2. Thermogravimetric-Differential Scanning Calorimeter (TG-DSC)

**Figure 8:**
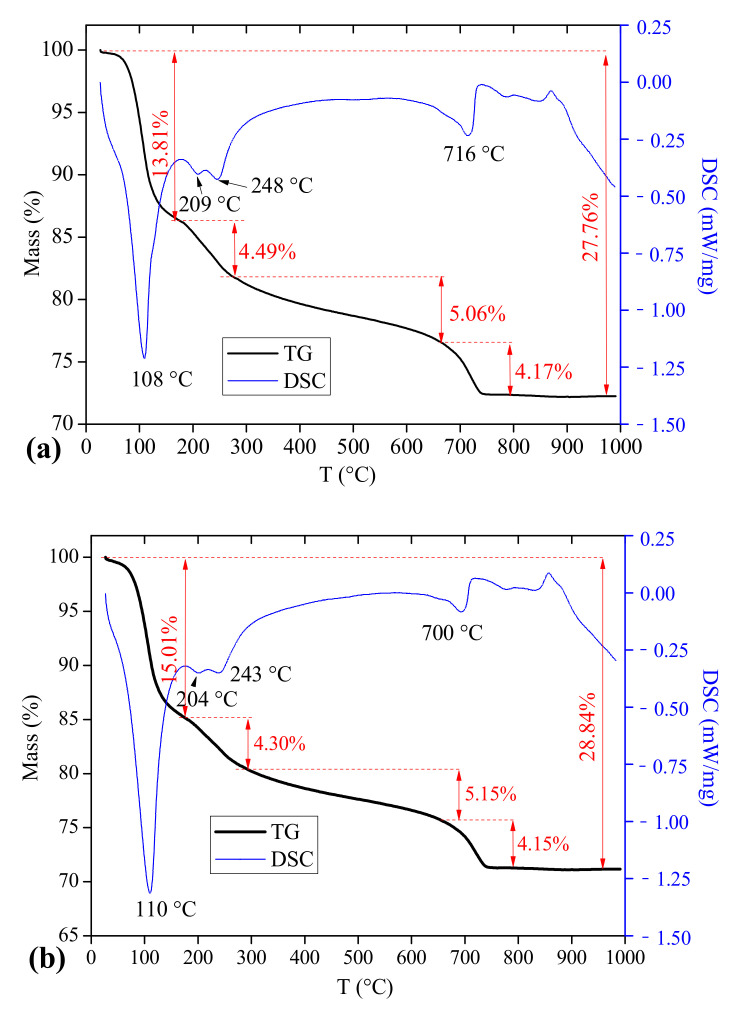

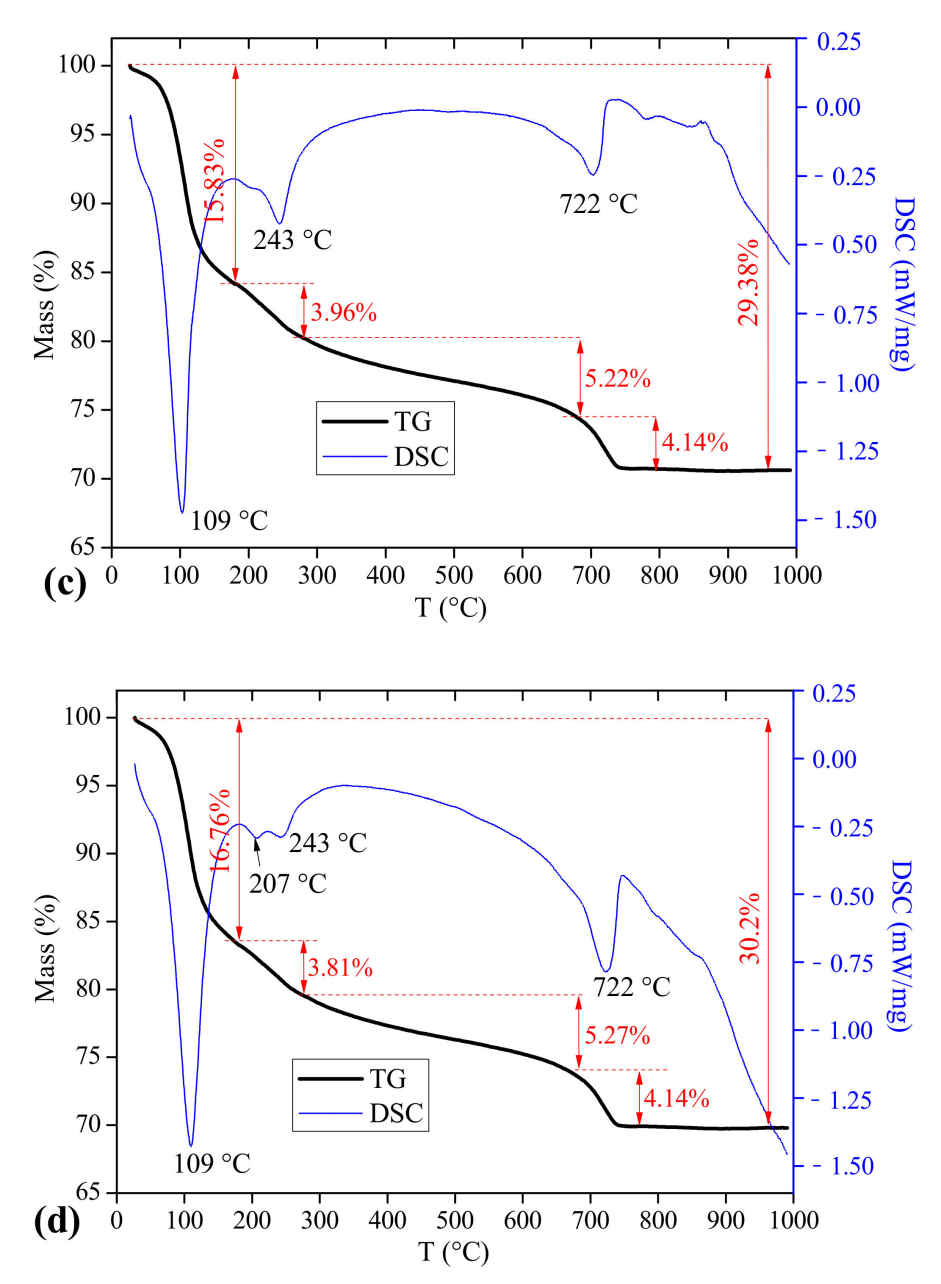
TG-DSC curves of CSAC-GGBFS mixtures with different FGDG content hydrated for 120 d. (**a**) 0%, (**b**) 3%, (**c**) 6% and (**d**) 9%.

**Figure 9:**
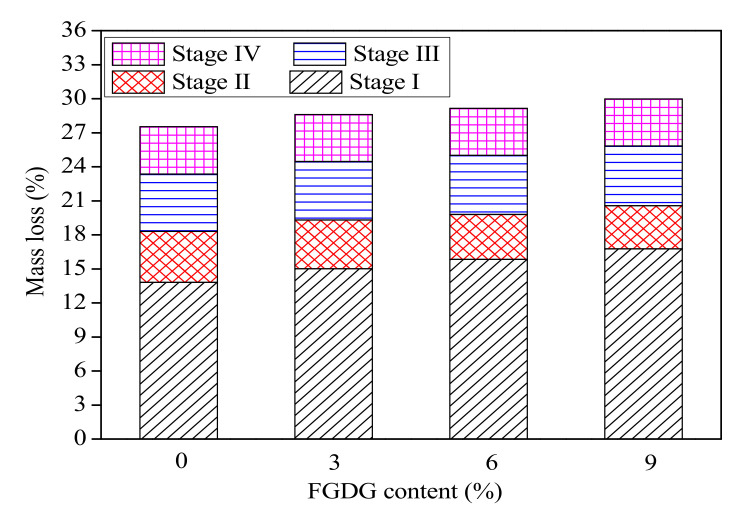
The mass loss at different stages for CSAC-GGBFS mixtures with different FGDG content hydrated for 120 d.

The TG-DSC curves of CSAC-GGBFS mixtures blended with different FGDG content hydrated for 120 d are shown in Figure 8. Similar to the description in reference [18], the mass loss of cement paste exposed to continuous heating is divided into four stages: (Ⅰ) <180 °C: the loss of inter-layer water in C–S–H gel and the dehydration of AFt; (Ⅱ) ~180 °C to ~280 °C: the dehydration of AFm and AH_3_; (Ⅲ) ~280 °C to ~660 °C: the dehydration of C–S–H gel and other hydration products; (Ⅳ) ~660 °C to ~740 °C: the decomposition of CaCO_3_. Figure 9 shows the mass loss of blended cement paste at four stages. Firstly, the mass loss of stage (Ⅰ) increases from 13.81% to 16.76% as the FGDG content increases from 0% to 9%; however, the mass loss of stage (Ⅱ) decreases slightly. These results illustrate that the addition of FGDG promotes the hydration of $C_{4}A_{3}\bar{S}$ to generate AFt and inhibits the transformation of AFt to AFm. Moreover, in the presence of CH and gypsum, AH_3_ can be consumed by the secondary hydration reaction (Equation (4)), and the amount of AFt increases [28]. The results also show that the mass loss of stage (Ⅲ) slightly increases with increasing FGDG content; that is to say, the amount of C–S–H gel increases. This proves that the existence of FGDG has a positive effect on activating the pozzolanic activity of GGBFS. Overall, as the FGDG content increases from 0% to 9%, the total mass loss of blended cement paste increases from 27.76% to 30.2% as the material is heated from room temperature to 1000 °C. This result confirms that the addition of FGDG promotes the hydration of CSAC-GGBFS cement paste, which also provides evidence that the mechanical strength improves with the increase in FGDG content.

**Figure 10:**
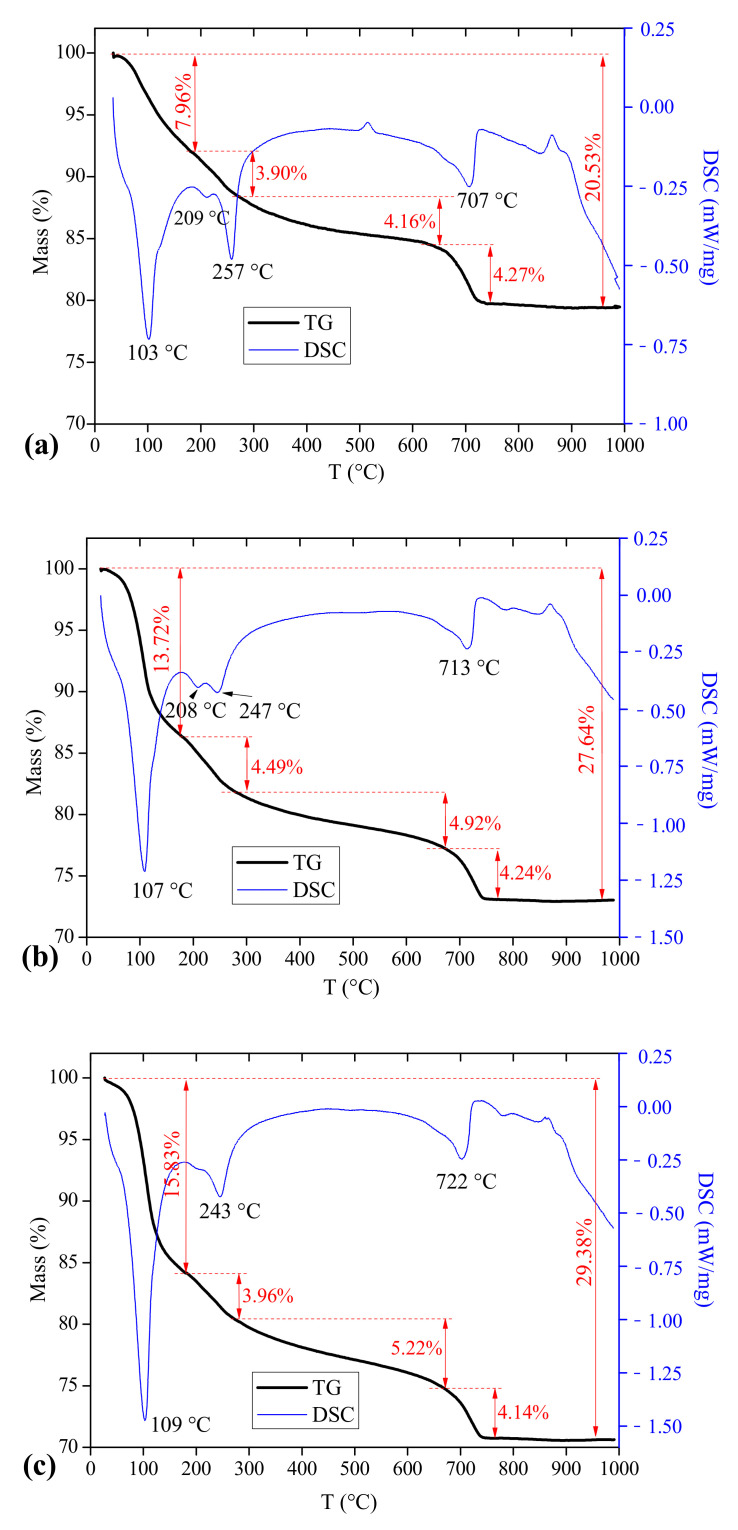
TG-DSC curves of the CSAC-GGBFS mixture with 6% FGDG hydrated for different time intervals. (**a**) 1 d, (**b**) 28 d and (**c**) 120 d.

**Figure 11:**
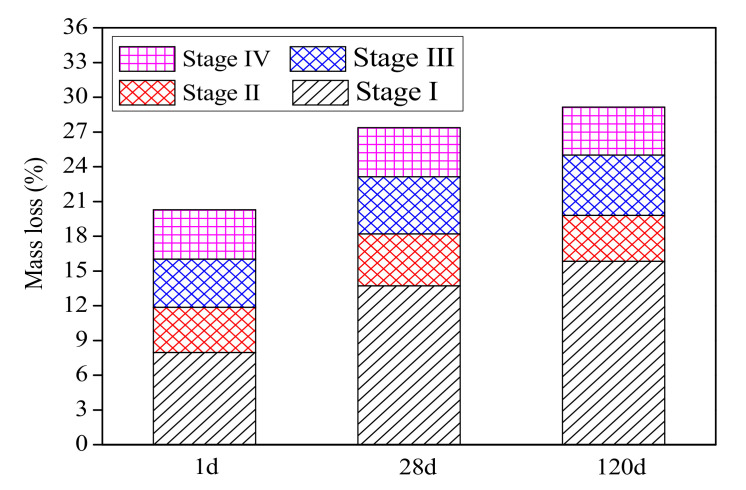
The mass loss at different stages for the CSAC-GGBFS mixture with 6% FGDG hydrated for different time intervals.

The TG-DSC curves and the mass loss at different stages for the CSAC-GGBFS mixture blended with 6% FGDG cured for 1 d, 28 d and 120 d are shown in Figure 10 and Figure 11, respectively. The mass loss of stage (Ⅰ) increases from 7.96% to 15.83% when the curing time increases from 1 d to 120 d, which indicates that the amount of AFt increases gradually. This result is consistent with the mineral phase analysis from XRD. The mass loss of stage (Ⅱ) increases at first when the curing time increases from 1 d to 28 d, and it decreases again at 120 d. This result indicates that the amount of AH_3_ is higher in the early hydration age, and it is then consumed by the hydration reaction (Equation (4)). In addition, the mass loss of stage (Ⅲ) increases continuously as the curing time increases from 1 d to 120 d; this indicates that the amount of C–S–H gel increases gradually. The total mass loss of cement paste increases from 20.53% to 27.64% as the curing time increases from 1 d to 28 d, and it reaches 29.38% at 120 d. According to the total mass loss, it can be seen that the hydration degree of CSAC-GGBFS-FGDG cement paste at 28 d is 94% of that at 120 d, which shows that the blended cement paste undergoes rapid hydration before 28 d.

#### 3.5.3. Scanning Electron Microscope (SEM)

**Figure 12:**
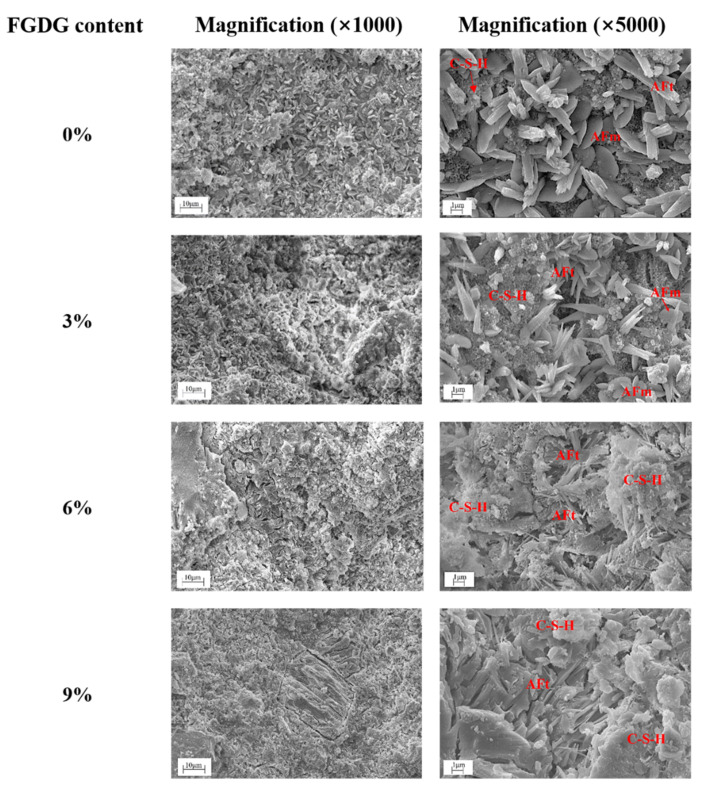
SEM images of CSAC-GGBFS mixtures with different FGDG content hydrated for 120 d.

Figure 12 shows the microstructure of CSAC-GGBFS mixtures with different FGDG content hydrated for 120 d. At low magnification (×1000), the compactness of hardened cement paste improves as the FGDG content increases from 0% to 6%, which is consistent with the analysis of pore structure. However, some micro-cracks appear when the FGDG content reaches 9% due to the increase in the AFt content. At high magnification (×5000), rod-shaped AFt, lamellar AFm and floccular morphology of C–S–H are interwoven together in the hardened cement paste without FGDG. However, with the addition of FGDG, the size of AFt noticeably decreases, and the amount of AFm decreases with increasing FGDG content. It can be seen that AFt is closely wrapped by a large amount of C–S–H gel to form a dense network structure when the FGDG content is 6%; however, the microstructure is cracked when the FGDG content reaches 9%, which is caused by the expansion of AFt. These results are direct evidence of the evolution of the pore structure and mechanical strength of hardened cement pastes with different FGDG content.

**Figure 13:**
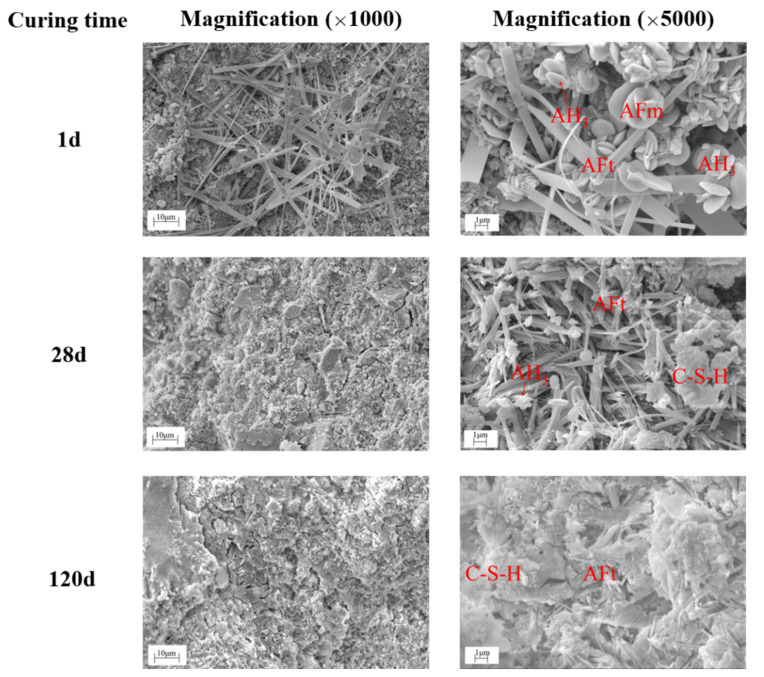
SEM images of the CSAC-GGBFS mixture with 6% FGDG hydrated for different time intervals.

The microstructure of the CSAC-GGBFS mixture blended with 6% FGDG hydrated for 1 d, 28 d, and 120 d is shown in Figure 13. It can be seen that baculiform-like AFt, flower-shaped AFm, oblate AH_3_ and unhydrated cement particles are loosely interwoven when the cement paste is hydrated for 1 d [29]. However, the amount of AFt noticeably increases at 28 d, and the particles are interlocked with each other. Furthermore, the AFt is compactly wrapped by a large amount of C–S–H gel when the pastes are hydrated for 120 d. It also can be seen that the morphology of AFt changes as the hydration age increases, which depends on the pH and sulfate concentration of the pore solution [26,30,31]. As shown in the SEM images, more AFt and C–S–H gel are generated in the blended cement paste when the hydration age increases from 1 d to 120 d, which improves the compactness of hardened cement paste and results in higher mechanical strength.

## 4. Conclusions

The effect of additional FGDG on the hydration of the CSAC-GGBFS system was investigated in detail. Some important conclusions are summarized as follows:

(1) The duration of the induction period for the blended cement paste is prolonged by the addition of FGDG. The early age hydration rate decreases as the FGDG content increases from 0% to 9%; however, the later age hydration is promoted.

(2) The addition of FGDG has little effect on the setting time of CSAC-GGBFS cement paste when its content increases from 0% to 9%, and the blended cement paste still exhibits the characteristic of rapid setting.

(3) The flexural and compressive strengths of CSAC-GGBFS mortar slightly decrease as the FGDG content increases from 0% to 9% at 1 d and 3 d. However, after 28 d, the addition of FGDG has a positive effect on the mechanical strength of the CSAC-GGBFS mixture. The compressive strength of mortar at 120 d can be improved by nearly 20% when the dosage of FGDG is 6%.

(4) The addition of FGDG can refine the pore size and reduce the total porosity of CSAC-GGBFS cement paste if its content is less than 6%. However, the pore size and total porosity increase when the FGDG content reaches 9%.

(5) The addition of FGDG promotes the hydration of CSAC-GGBFS cement paste and increases the presence of hydration products, which further improves the mechanical strength of mortar at the later age. However, the FGDG content should not exceed 6%, or some micro-cracks appear in the cement paste. An appropriate increase in gypsum content benefits the hydration and mechanical strength of CSAC blended with SCM.

## Figures and Tables

**Table 1:** Chemical compositions of raw materials (wt %) [18].

Composition	CSAC	GGBFS	FGDG
CaO	47.88	51.65	43.17
Al_2_O_3_	20.83	12.85	1.16
SO_3_	17.37	2.80	47.94
SiO_2_	7.49	25.56	3.45
Fe_2_O_3_	2.03	1.11	0.48
MgO	1.91	2.95	2.27
TiO_2_	1.06	1.17	-
K_2_O	0.82	0.42	0.23
Na_2_O	0.15	0.26	0.17
Cl	0.12	0.08	0.56
SrO	0.1	0.15	0.03
F	0.08	-	0.50
P_2_O_5_	0.06	0.10	0.02

**Table 2:** Mix proportions (wt %).

No.	CSAC	GGBFS	FGDG	Water
1	80	20	0	50
2	77.6	19.4	3	50
3	75.2	18.8	6	50
4	72.8	18.2	9	50

## Data Availability

The data presented in this study are available on request from the corresponding author.
